# Intracompartmental 3D Printing of Enzymatically Active Organelle Mimics

**DOI:** 10.1021/acsnano.5c14167

**Published:** 2025-11-06

**Authors:** Yiğitcan Sümbelli, Anna C. Jäkel, Madelief A. M. Verwiel, Nadia A. Erkamp, Alexander F. Mason, Friedrich C. Simmel, Jan C. M. van Hest, Alexander B. Cook

**Affiliations:** † Department of Biomedical Engineering, Institute for Complex Molecular Systems (ICMS), 3169 Eindhoven University of Technology, Eindhoven 5600 MB, Netherlands; ‡ Department of Chemical Engineering and Chemistry, Institute for Complex Molecular Systems (ICMS), Eindhoven University of Technology, Eindhoven 5600 MB, Netherlands; § Physics of Synthetic Biological Systems, Department of Bioscience, School of Natural Sciences, Technical University of Munich, Garching 85748, Germany; ∥ School of Science, Molecular Horizons, University of Wollongong, Wollongong, New South Wales 2522, Australia

**Keywords:** artificial cell, artificial organelle, 3D printing, photopolymerization, compartmentalization, coacervate

## Abstract

Introducing subcellular structures in artificial cells is a key step in mimicking the structure and role of organelles, which are instrumental in compartmentalizing cellular reaction networks. Despite the variety of strategies to include subcellular features within artificial cell models, achieving spatial and morphological control over these compartments remains challenging. In this study, we engineered 3D-printed subcellular compartments within terpolymer-stabilized coacervate-based artificial cells. Coacervate-forming charged polymers were functionalized with methacrylate moieties, enabling the fabrication of a variety of architectures within droplets through photoinitiated radical polymerization. The addition of a Ni-NTA functional methacrylate monomer to the coacervates led to its sequestration upon polymerization in these subcellular regions. As a result, the compartments were able to uptake and concentrate His_6_-tagged mTurquoise and β-galactosidase protein cargo molecules, despite the increase in viscosity that was induced upon polymerization. Following this affinity-based interaction approach, we demonstrated the region-specific localization of an enzymatic reaction within the artificial cells.

## Graphical abstract

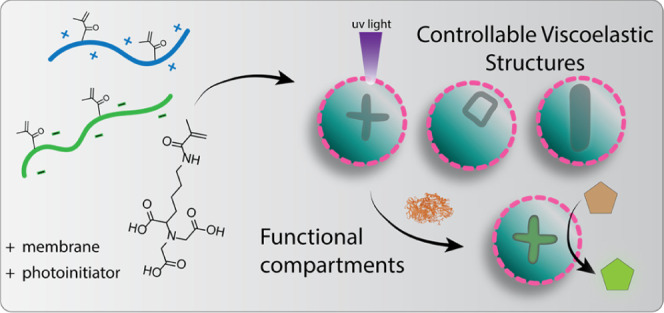

## Introduction

Artificial cell research is a developing field focused on studying the fundamental functions of native cells by employing various engineering approaches. The primary goal of the field can be seen as creating fully functioning mimics at various levels of complexity, ranging from single-cell to multicellular tissue-like structures that can self-maintain in vitro and, potentially, in vivo.^1–3^ Challenges, however, are not only to be found in the orchestration of intercellular processes. Intracellular organization, as specifically observed in eukaryotic cells, is equally essential for regulating biochemical pathways to achieve life-like behavior.^4–7^

Specialized subcellular interactions in natural cells take place within discrete compartments called organelles, and introducing such compartments into artificial cells is essential for achieving higher-order control over reactions and communication pathways in life-like systems. Mimicking the natural compartmentalized organization within synthetic systems, however, requires precise control over the physical and chemical properties of the artificial organelles. Over the years, various strategies have been proposed to incorporate subcellular compartments into both native and artificial cells.^8,9^ Embedding preformed compartments is one approach that maintains the stability of the encapsulated particles, or compartments, during incorporation into artificial cells. In this regard, polymersomes,^10^ liposomes,^11,12^ and coacervates,^13^ have been used to mimic the hierarchical organization within artificial cells. Another strategy for incorporating compartments into artificial cell models involves the spontaneous formation of subcellular structures in response to a triggering stimulus.^14^ Inducing liquid–liquid phase separation within complex coacervates by tuning electrostatic interaction strength,^15,16^ forming intracellular coacervates by the expression of phase-separating proteins,^17^ trapping them within aqueous two-phase systems during formation,^18^ or inducing self-assembly of polymeric structures^19^ are some approaches to mimic the compartmentalized complexity of natural cells. These strategies all offer various advanced cellular features, ranging from increased structural stability^20–23^ and morphological transformation,^24^ to the localization of specific cellular functions.^25,26^

The majority of compartmentalization approaches, however, fail to offer tunability for the chemical composition of the compartments, precise spatial placement within the artificial cell model, or temporal control of the reactions within the compartments. Recently, light-based approaches have been reported to generate complex subcellular compartments in situ, overcoming some of the limitations of the above strategies.^27–30^ Using a high-resolution additive manufacturing technique to integrate hydrogel structures into vesicle artificial cell models resulted in an engineered subcellular structure with tunable physical and chemical properties, as well as with spatiotemporal control for integrating biochemical reactions. Yet, despite the precise structural control, the organelles were incorporated into the lumen of a vesicle, which lacks the crowdedness of the cytoplasm and, as such, emulates the natural environment less closely in which organelles are typically positioned.

To overcome this limitation, we implemented a light-based 3D printing approach to engineer discrete functional regions within complex coacervate-based artificial cells. The applicability of using coacervates as artificial cells and organelles has previously been discussed in several studies.^31–35^ Our complex coacervate subcellular printing approach ensured the engineering of 3D printed regions (3DPRs) with high resolution, while maintaining externally controlled subcellular organization in an inherently crowded environment, rather than spontaneously forming subcellular structures. We demonstrated the ability to form precise architectures by printing a variety of micrometer-sized constructs and characterized fluidity using fluorescence recovery after photobleaching (FRAP) experiments. Furthermore, integrating an affinity-based interaction (His-tag/Ni-NTA) within the 3DPRs enabled the study of highly localized subcellular enzymatic reactions. This investigation allows the construction of artificial organelle-mimicking structures with precise patterned spatial control, which enables a further sophistication of the artificial cell’s functional architecture.

## Results and Discussion

### Establishment of Radical-Based Cross-Linking of Amylose Coacervates

The artificial cell platform was constructed based on our previous work, by the phase separation of charged amyloses and subsequent stabilization with a membrane-forming terpolymer, poly­(ethylene glycol)-poly­(caprolactone-gradient-trimethylene carbonate)-poly­(glutamic acid) (PEG-p­(CL-*g*-TMC)-pGlu).^36^ We introduced photopolymerizable methacrylate groups onto the amyloses via the methacrylic anhydride modification of amylose hydroxyl groups. Figure 1 provides an overview of the experimental setup. After the spontaneous formation of terpolymer-stabilized complex coacervates, either the entire droplets were cross-linked with UV irradiation, or 3DPRs as artificial organelle-mimics were fabricated via the 405 nm laser line of a confocal laser scanning microscope (CLSM) (in the presence of photoinitiator lithium phenyl-2,4,6-trimethylbenzoylphosphinate (LAP)).

**Figure 1:**
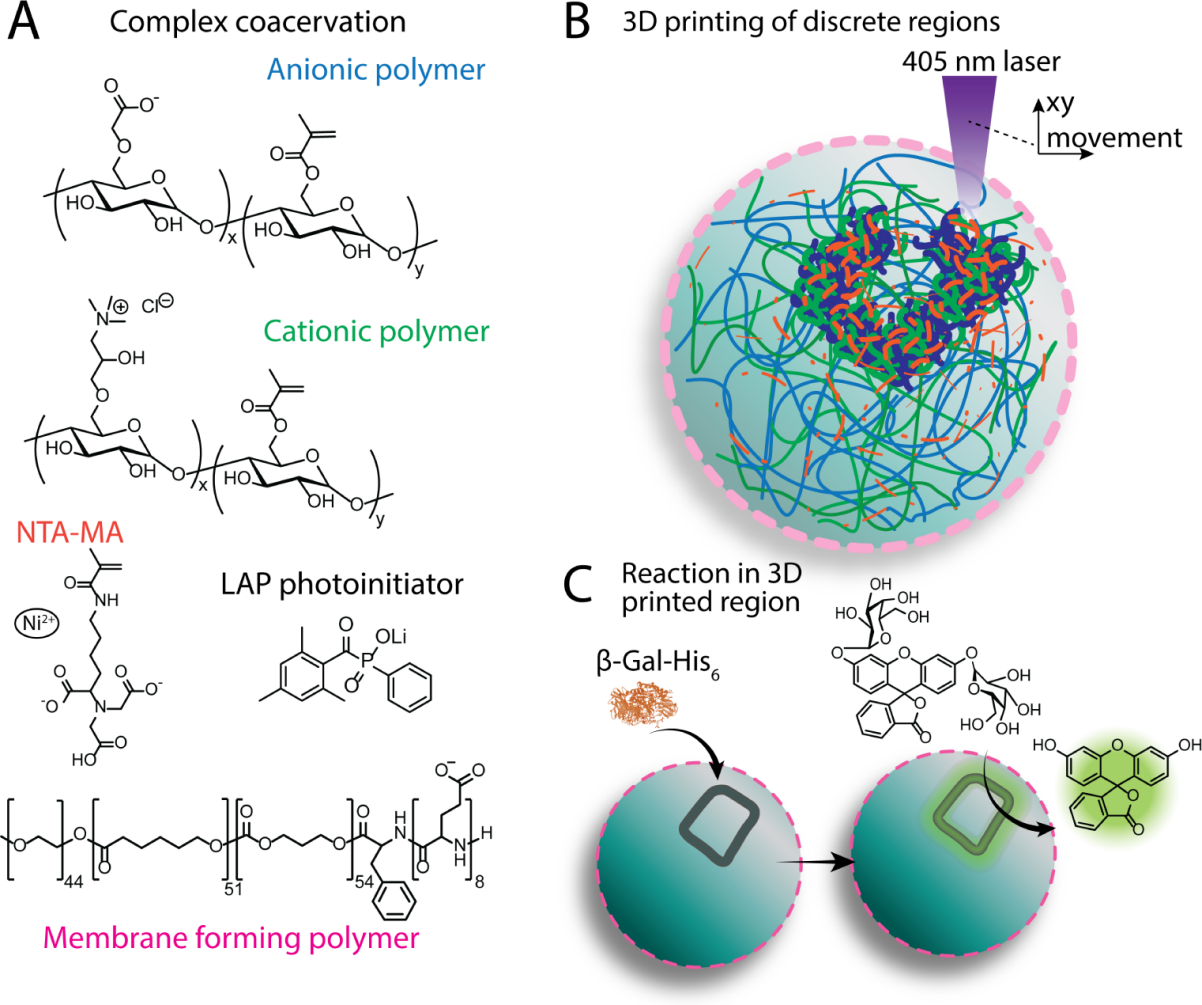
Overview of the polymers used for photopolymerizable complex coacervate formation, subsequent 3DPR printing, and the model enzymatic reaction process. (A) Chemical structures of the anionic polymer methacrylated carboxymethyl amylose (CM-Am-MA), cationic polymer methacrylated quaternary amine amylose (Q-Am-MA), methacrylated nitrilotriacetic acid (NTA-MA), photoinitiator lithium phenyl-2,4,6-trimethylbenzoylphosphinate (LAP), and synthetic membrane-forming triblock copolymer (poly­(ethylene glycol)-poly­(caprolactone-gradient-trimethylene carbonate)-poly­(glutamic acid) (PEG–PCL-*g*-TMC-pGlu)), (B) the 3D printing of defined structures within the coacervates by the 2-axis movement of a 405 nm laser, and (C) the localized production of fluorescent molecules via the enzymatic reaction between β-galactosidase and fluorescein-di-β-d-galactopyranoside (FDG).

### Effect of Coacervate Cross-Linking and Cargo Molecular Weight on Partitioning Behavior

First, the dynamicity and uptake behavior of the coacervate samples were investigated as a function of polymerization time (1, 5, or 10 min at 100% laser intensity). The coacervates were cross-linked in their entirety in the absence of methacrylated nitrilotriacetic acid (NTA-MA) moieties, while maintaining their spherical droplet form (Figures S1–S3). Dynamicity was studied using fluorescein isothiocyanate-labeled dextran 4 kDa (FITC-Dex (4 kDa)) as a cargo molecule, which was added to the cross-linked samples. Fluorescence recovery after photobleaching (FRAP) measurements showed a decline in cargo mobility, correlating with increased cross-linking time (Figures 2B, S4 and S5). Calculated diffusion constants from the FRAP measurements further supported the hindered diffusion of the cargo molecules for the extended cross-linking periods (Figure S6).

**Figure 2:**
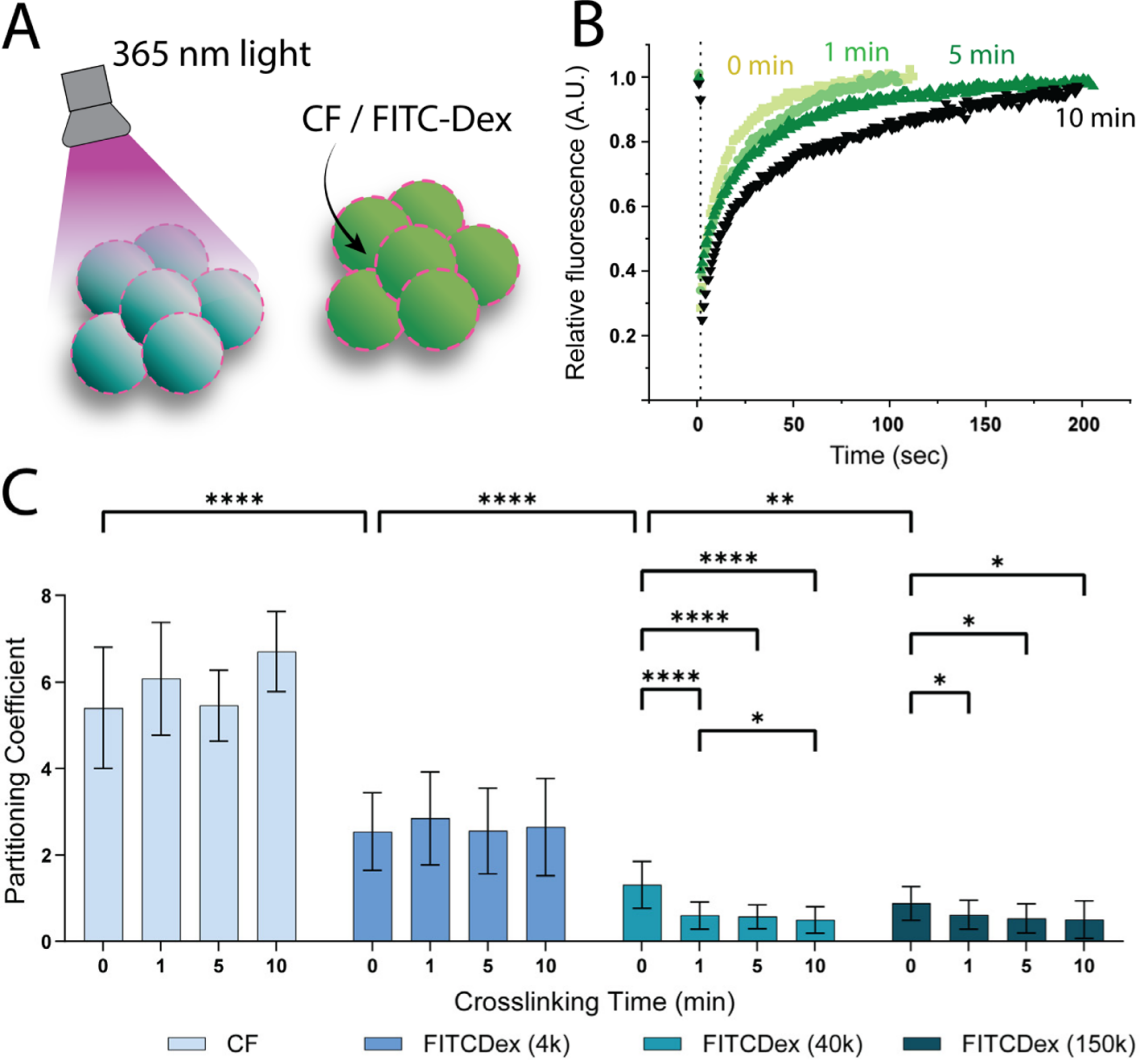
Cargo sequestration behavior of artificial cells and their cross-linked counterparts. (A) Schematic illustration for the bulk cross-linking approach. (B) Cross-linking resulted in decreased fluorescence recovery after photobleaching (FRAP), (C) the effect of cargo molecular weight and cross-linking on partitioning. The combined overview of partitioning coefficients, grouped by cargo type, across different cross-linking times, illustrates the collective effect on cargo sequestration for the higher molecular weights. Bars represent mean ± SD of individual droplet measurements. Significant differences are visualized on bar plots using asterisks to indicate p-value ranges (ns: *p* > 0.05, *p ≤ 0.05, **p ≤ 0.01, ****p ≤ 0.0001. Only statistically significant results were shown on the graph) (*n* = 35 per condition across 3 independent experiments).

To investigate factors that affect cargo sequestration, partitioning coefficients were measured for dextrans of varying molecular weights (FITC-Dex 4 kDa, 40 kDa, 150 kDa, and the low molecular weight carboxyfluorescein (CF)) and at four different cross-linking times. A two-way ANOVA test using the Geisser-Greenhouse correction, followed by Tukey’s multiple comparisons post hoc analysis was used to analyze the results from 3 experiments conducted independently by using different samples. For cargo molecular weights lower than 40 kDa, the partitioning was primarily determined by the molecular weight regardless of the cross-linking time. When the cargo molecular weight was equal to or higher than 40 kDa, the cross-linking time took a shared role in affecting the partitioning. This phenomenon can be seen in the FITC-Dex (40 kDa) and (150 kDa) samples when the cross-linking time was increased to 1, 5, and 10 min (Figure 2C). The significant decrease in partitioning can be explained by the size exclusion effect on cargo molecules due to a more compact network density upon longer cross-linking times, which mainly affects the higher molecular weights, thanks to their larger hydrodynamic radius (Tables S1 and S2).

### Internal Photopatterning of Coacervate Artificial Cells

Next, polymerization in the absence of NTA-MA moieties was conducted in a well-defined region within the coacervate artificial cell in order to construct 3D printed regions (3DPRs), using the laser of the CLSM. The printing accuracy and the uptake of cargo molecules within these 3DPRs were subsequently investigated (Figure 3A). Different shapes were successfully printed within similarly sized coacervates using the same CLSM irradiation settings (Figure 3B). Following this, cargo molecules with different molecular weights were added to the samples that contained 3DPRs (Figure 3C,D). As expected, we observed that the sequestration efficiency of cargo molecules in the 3DPRs was reduced compared to the non-cross-linked regions of the coacervates. Although the cargo with a small molecular weight, CF, could quickly diffuse through the coacervate (Figure 2C), its uptake dynamics was hindered within the cross-linked regions of the same coacervates. The altered diffusion rates can explain this behavior, resulting from the increased polymeric network density in the locally cross-linked areas, thanks to the local photopatterning approach. Even though the penetration capability of 365 nm light, which was used for the bulk cross-linking approach, was higher than that of the 405 nm laser, the local photopatterning approach resulted in denser and more confined cross-linked networks in shorter irradiation times, due to the higher localized energy density.

**Figure 3:**
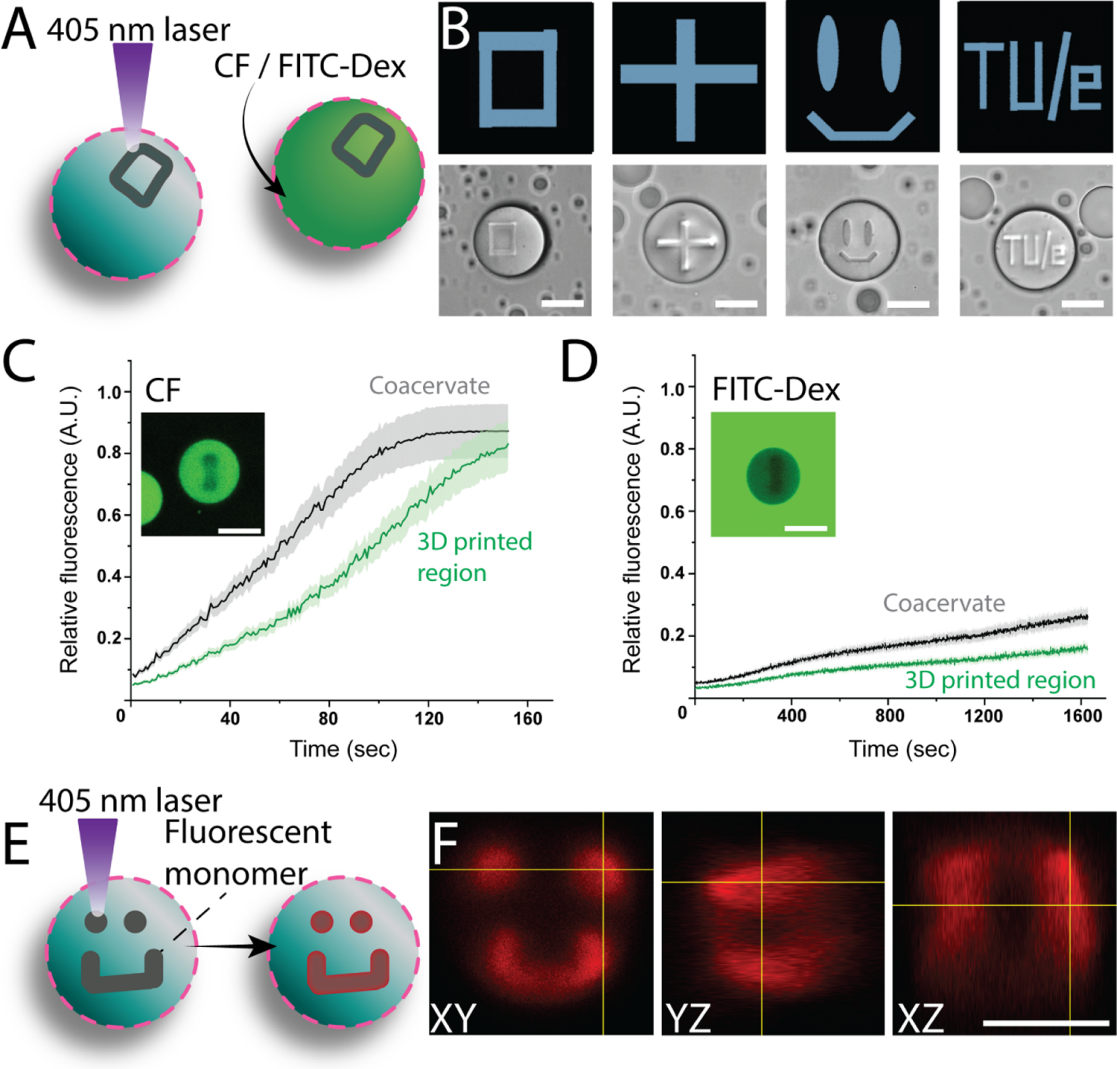
Constructing 3DPRs using a confocal laser printing approach offers high structural fidelity within coacervates. (A) Schematic of CLSM based 3DPR printing (B) photopatterned structures are formed. (Scale bars: 25 μm). The uptake dynamics of (C) carboxyfluorescein (recorded for 2.5 min) and (D) FITC-Dex (40 kDa) (recorded for over 24 h) is diminished in the 3DPR compared to the (non-cross-linked) artificial cell within a 25 min measurement time. (E) Schematic of 3DPR fabrication by including a fluorescent monomer, acryloxyethyl thiocarbamoyl rhodamine B. (F) Orthogonal views of the CLSM micrographs show the distribution of the 3DPR within the droplet, while displaying high printing resolution. (Scale bar: 25 μm).

Furthermore, the volumetric size and distribution of the 3DPRs were analyzed. The polymerized network was visualized by including a photopolymerizable fluorescent monomer, acryloxyethyl thiocarbamoyl rhodamine B, RhBA (Figures 3E and S7). The orthogonal views of the 3DPR show the polymerized regions emitting a higher fluorescent signal due to incorporation of the RhBA into the polymer network (Figure 3F). The 3-dimensional structure of the 3DPR did not alter the structural stability of the droplets despite covering a large volume within the photopolymerizable coacervate (Figure S8).

### Directed Protein Uptake in 3DPRs

To compensate for the limited uptake in the 3DPRs and to effectively equip them with biomolecular cargo to better mimic natural organelle function, NTA-MA moieties were added to the coacervates and copolymerized upon cross-linking. Due to the polymerization process, an enrichment of the NTA moiety in the 3DPR phase was achieved. In presence of Ni^2+^ His_6_-tagged proteins were now expected to be locally sequestered (Figure 4A). To demonstrate this behavior, the fluorescent protein mTurquoise-His_6_ was introduced, which indeed accumulated in the printed regions, regardless of mTurquoise-His_6_ having a higher molecular weight (monomer *m*_w_: 26.9 kDa) than CF (376.3 g/mol) (Figure 4B). Furthermore, the fluorescent protein was specifically localized in the 3DPR after a sufficient incubation period (Figures 4C and S9). Despite the possible presence of non-cross-linked NTA-MA moieties in the nonprinted region, the high local concentration of the NTA affinity tags and the resulting multivalent interactions were responsible for the observed localized uptake.

**Figure 4:**
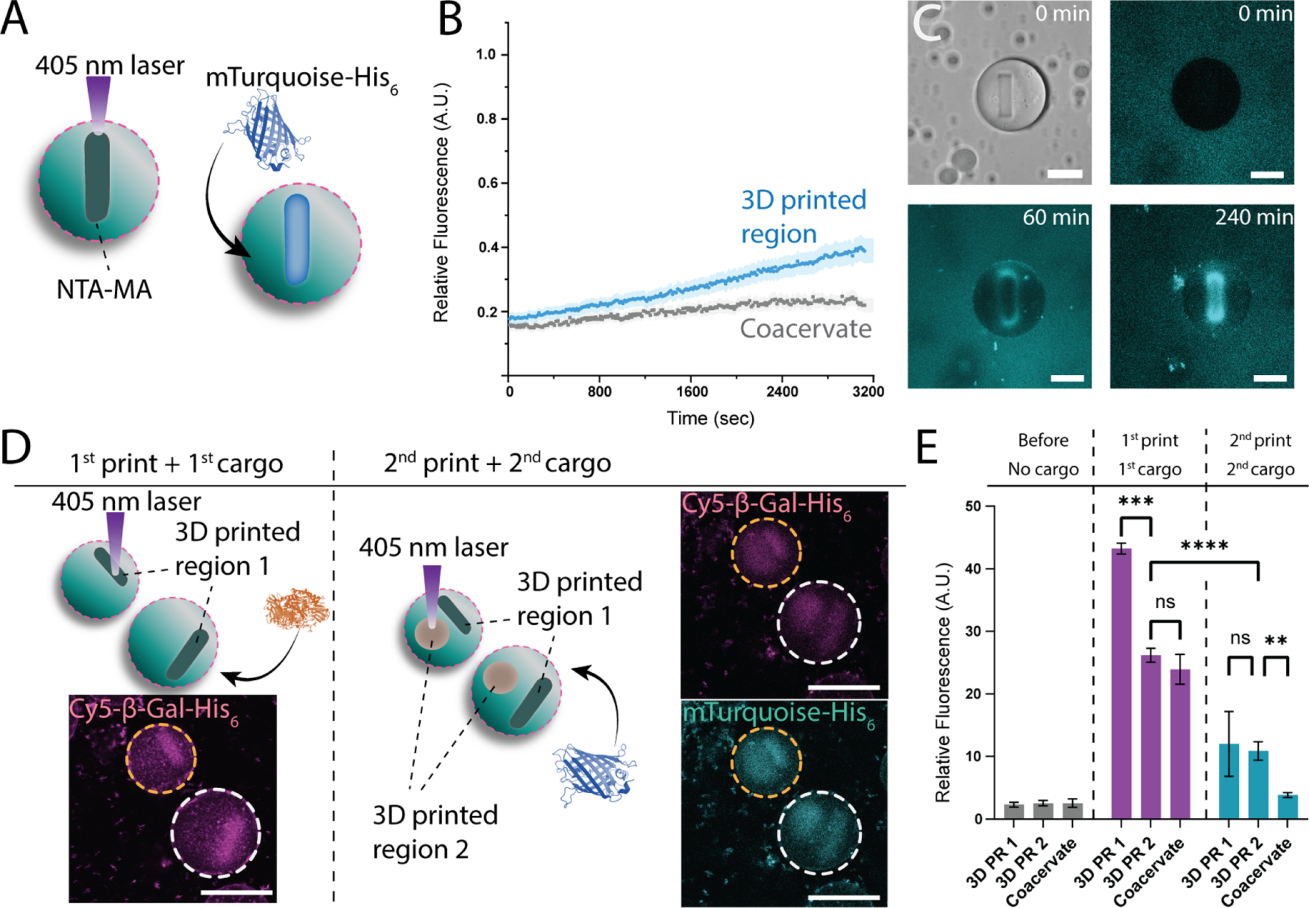
Localized uptake of cargo molecules can be achieved by NTA-His tag interactions. (A) Schematic of protein sequestration in printed 3DPR regions. (B) Fluorescence intensity increase of mTurquoise-His_6_ being locally sequestered within the 3DPR, via the interaction between the histidine residues and the NTA moieties, and (C) localization of His_6_-tagged fluorescent proteins in the 3DPR at different time points can be observed by CLSM (Scale bars: 25 μm). (D) The sequential printing and cargo addition progress in 4 steps. Upon the printing of the first 3DPRs, the localized uptake of the first cargo can be followed by CLSM. Next, the second 3DPRs are fabricated, and the subsequent second cargo addition can be seen by CLSM (scale bars: 25 μm), (dashed lines show the separate coacervates. Note the movement of each droplet due to liquid flow in between each step), and (E) the uptake behavior of both cargo molecules is quantified for both 3DPRs, respectively. The higher signal intensities showcase the localization of the cargo molecules within the 3DPRs. The decrease in the fluorescence intensity after the second print was observed due to the partial bleaching caused by the printing process. Bars represent mean ± SD of individual droplet measurements. Significant differences are visualized on bar plots using asterisks to indicate *p*-value ranges (ns: *p* > 0.05, *p ≤ 0.05, **p ≤ 0.01, ****p ≤ 0.0001) (*n* = 5 per condition).

Next, a sequential 3DPR fabrication and the subsequent cargo uptake behavior were realized. 3DPRs were fabricated in the presence of NTA-MA moieties, and cargo molecules were locally sequestered within the 3DPRs. First, a rectangular-shaped 3DPR was fabricated, and the first cargo, Cy5-β-Gal-His_6,_ was added to the sample. After an overnight incubation in a dark and sealed environment, the second 3DPR was fabricated in a circular shape, next to the first 3DPR, and the second cargo, mTurquoise-His_6_, was added. After an overnight incubation, cargo uptake behavior was measured (Figure 4D). A repeated-measures two-way ANOVA test with the Geisser-Greenhouse correction, combined with Tukey’s multiple comparisons test, was applied to analyze the results. Localized uptake behavior was observed after both cargo addition steps, despite the second printing process causing a drop in the signal intensity of the first cargo due to partial bleaching during prolonged laser exposure (Figure 4E). The monoselective nature of Ni-NTA—His-tag affinity limits the cargo selectivity. Future research could incorporate a second orthogonal cargo uptake mechanism, which would improve selectivity for multiple cargo systems.

### Biocatalytic Reactions in 3DPRs

After establishing the selective affinity-based uptake of His-tagged proteins, a His-tagged enzyme was exploited to implement a biocatalytic reaction in the organelle-mimicking 3DPRs (Figure 5A). His_6_-tagged beta-galactosidase (β-Gal-His_6_) was indeed sequestered by the coacervates (Figure 5B), and the accumulation of the cargo was localized in the 3DPRs (Figure 5C). The enzyme’s profluorescent substrate probe fluorescein di-β-d-galactopyranoside (FDG) was subsequently added to the artificial cells (Figure 5D). The fluorescent product was formed in the 3DPRs where the enzyme was concentrated (Figures 5E and S10). Interestingly, product formation was seen not only in the 3DPRs but also in the coacervates that did not contain printed regions or NTA moieties (Figure 5F). This result was consistent with the fast diffusion characteristics and high uptake behavior of the low molecular weight cargo molecules where sequestration was observed throughout the coacervate population (Figure 3C). The formed product’s low molecular weight (330 Da) allows it to diffuse through the whole coacervate despite the densely cross-linked 3DPRs. However, the high local concentration of the enzyme in the 3DPRs resulted in higher product formation compared to the coacervates that did not contain affinity-based interactions. After a 60 min incubation time, the 3DPR still displayed the highest fluorescence compared to the control samples.

**Figure 5:**
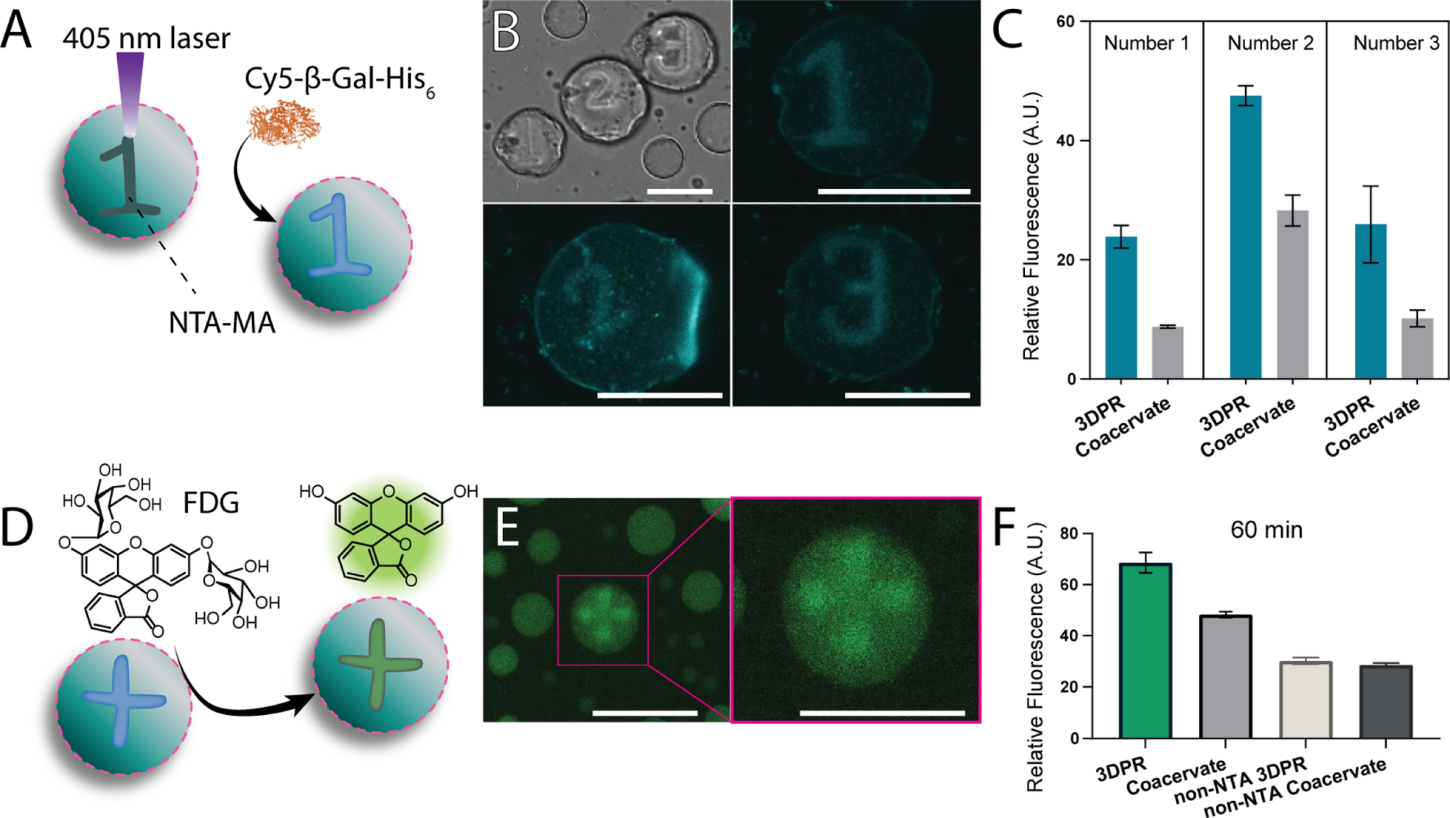
3DPR-confined enzyme activity inside artificial cells. (A) The schematic illustrates the uptake of beta-galactosidase (β-Gal-His_6_) into coacervates. (B) CLSM micrographs showing the enzyme uptake within the coacervates (scale bars: 25 μm), and (C) the enzyme accumulation localized in the 3DPRs. (D) The schematic illustrates the subsequent treatment with the profluorescent substrate fluorescein di-β-d-galactopyranoside (FDG), generating fluorescent signal upon product formation. (E) CLSM micrograph showing the enzymatic formation of fluorescent molecules within the 3DPR after 60 min (scale bar: 25 μm) (F) intensity quantification of end point fluorescent signal after 60 min reaction. The analysis shows that the reaction rate is higher in NTA-containing 3DPRs than in cross-linked regions without NTA.

## Conclusions

Spatiotemporal control over biochemical reactions in specialized organelle compartments is a fascinating feature of native cells. Implementing such behavior in artificial organelles with precise and tunable architectures has been challenging due to the lack of effective construction methods. With our highly controlled light-based 3D printing approach, we have developed a new strategy to engineer designer subcellular structures with controllable diffusivity and selective cargo sequestration. Quaternised-amylose and carboxymethyl-amylose were functionalized with methacrylate moieties, enabling 3DPRs with different morphologies to be cross-linked inside complex coacervates. Cross-linking of amyloses reduced the uptake of cargo molecules of all molecular weights, but particularly higher molecular weight FITC–dextrans. However, by also including NTA-methacrylate during photopatterning, the compartments were able to effectively uptake His_6_-tagged mTurquoise and β-galactosidase, despite their large molecular weights. We showed that different 3DPRs within the same coacervate can locally sequester sequentially added multiple cargo molecules. Yet, the limiting selectivity of the Ni-NTA—His-tag affinity prevents us from controlled shuttling of different cargo molecules to different 3DPRs within the same coacervate. Finally, we also demonstrated the compartment-specific localization of an enzymatic reaction within the artificial cells. Leveraging such precise control over subcellular organization in artificial cell communities can be used for the engineering of hybrid tissues, where different populations interact through communication mechanisms working at both meso- and macroscales.

## Materials and Methods

### Materials

Methacryloyl chloride, 6-amino-2-[bis­(carboxymethyl)­amino]­hexanoic acid, triethyl amine, NaOH pellets, 3-chloro-2-hydroxypropyltrimethylammonium chloride solution (60 wt % in water), chloroacetic acid, methacrylic anhydride, carboxyfluorescein, fluorescein di-β-d-galactopyranoside (FDG), fluorescein isothiocyanate (FITC)-dextran (4 kDa, 40 kDa, 150 kDa), lithium phenyl-2,4,6-trimethylbenzoylphosphinate (LAP), were purchased from Merck KGaA. Amylose (12–16 kDa) was purchased from Carbosynth. Terpolymer mPEG-p­(CL-*g*-TMC)-pGlu was prepared and purified as previously reported.^37^ Acryloxyethyl thiocarbamoyl rhodamine B was purchased from Merck KGaA. Recombinant hexahistag β-galactosidase, from *Escherichia coli,* (β-Gal-His_6_), was purchased from Abcam. Cyanine5 NHS ester (Cy5-NHS) was purchased from Lumiprobe. Cy5 labeled β-Gal-His_6_, was prepared following a previously reported procedure.^38^ Briefly, Cyanine5 NHS ester was incubated with β-Gal-His_6_ in PBS at pH 7 for 24 h, and dialyzed against PBS for 48 h to remove unconjugated dye. His-mTurquoise was expressed in *E. coli* *BL21*(*D3*) cells from a plasmid under the control of a T7 promoter overnight at 16 °C. After harvesting, cells were lysed with lysozyme (1 mg/mL), DNase, PMSF, and benzamidine (1 mM each), followed by sonication and centrifugation. The His_6_-tagged protein was purified using a HisTrap column on an ÄKTA system with imidazole gradient elution, and eluted fractions were pooled, buffer exchanged into storage buffer (50 mM Tris–HCl pH 8.0, 100 mM NaCl, 1 mM DTT, 1 mM EDTA, 50% glycerol), and stored at −80 °C. All other chemicals and solvents were purchased from Merck KGaA.

### Characterization

^1^H nuclear magnetic resonance (NMR) spectroscopy was conducted on a Bruker Avance 400 MHz spectrometer in either deuterated chloroform (CDCl_3_) or deuterated water (D_2_O). Brightfield microscopy was conducted with a Leica TCS SP8, and the images were analyzed with FIJI (ImageJ). Size distributions of coacervates were determined using standard ImageJ functions. The droplets were selected using manual thresholding; droplets located on the image edge were excluded from the analysis. A minimum of 80 droplets were analyzed per sample. Coacervates were further imaged with a Leica TCS SP8 (63× water immersion objective) confocal laser scanning microscope (CLSM) equipped with laser lines of 405, 488, 552, and 638 nm; the pinhole was set to 1 Airy Unit. An 18-well μ-slide (Ibidi) was used to image coacervate suspensions. A laser set at 488 nm and emission of 510–580 nm was used to image the fluorescein-derived fluorophores. Images were analyzed using FIJI (ImageJ).

### Amylose Q- and CM-Modification

Amylose Q- and CM-modifications were conducted following the previously published method.^37^ Briefly, quaternized amylose (Q-Am) was prepared by dissolving 1.5 g of amylose and 4.9 g of NaOH in 30 mL Milli-Q at 40 °C. After the complete dissolution of the amylose, 13 mL of 3-chloro-2-hydroxypropyltrimethylammonium chloride solution (60 wt % in water) was added dropwise to the stirring reaction mixture, which was subsequently left to react overnight. Carboxymethylated amylose (CM-Am) was prepared by dissolving 1.5 g of amylose and 4.9 g of NaOH in 30 mL Milli-Q at 40 °C. After the complete dissolution of the amylose, 4.51 g of chloroacetic acid was added, and the reaction mixture was stirred overnight. Both reaction mixtures were neutralized with acetic acid and precipitated in cold ethanol. The resulting precipitate was redissolved in Milli-Q water and dialyzed against water (3.5 kDa MWCO) before lyophilization. ^1^H NMR characterization data are presented in Figures S11 and S12. The degree of substitution was calculated with ^1^H NMR spectroscopy to be 1.24 for Q-Am and 0.43 for CM-Am.

### Amylose Methacrylation

CM-Am or Q-Am were modified with methacrylate groups following the same procedure (Figures S13 and S14); the procedure for Q-Am is described here as an example. The protocol is a variation on previously reported procedures.^39^ Q-Am (50 mg, 0.162 mmol) was dissolved in 4 mL NaOH (pH 9.5), and an excess of methacrylic anhydride (250 mg, 1.62 mmol), compared to the amylose average repeat unit weight, was added. After stirring at room temperature for 24 h, the reaction mixture was precipitated in cold ethanol. The resulting precipitate was washed with ethanol, redissolved in Milli-Q water, and dialyzed against water (3.5 kDa MWCO) before lyophilization. ^1^H NMR characterization data are presented in Figures S15 and S16. The degree of substitution was calculated with ^1^H NMR spectroscopy to be 0.2 for Q-Am (40 mg, ∼80% yield) and 0.1 for CM-Am (36 mg, ∼75% yield).

### Synthesis of 2,2′-((1-Carboxy-5-methacrylamidopentyl)­azanediyl)­diacetic Acid (NTA-MA)

In a round-bottom flask equipped with a magnetic stir bar, 6-amino-2-[bis­(carboxymethyl)­amino]­hexanoic acid (493 mg, 1.88 mmol) in 15 mL of NaOH solution (0.5 M) was cooled to 0 °C in an ice bath. Methacryloyl chloride (216 mg, 2.07 mmol) was dissolved in 7.5 mL of toluene and added dropwise over 1 h with stirring. The reaction proceeded for approximately 4 h, with continued stirring after the addition of methacryloyl chloride (Figure S17). Upon completion, the crude reaction mixture was concentrated via rotary evaporation. The product was purified by passing an aqueous solution of the crude product over Dowex 50WX8 to remove ions and lyophilized to obtain NTA methacrylate as a clear, viscous oil (576 mg, 93% yield). Structure and purity were confirmed by ^1^H and ^13^C NMR spectroscopy (Figures S18 and S19). ^1^H NMR (D_2_O): δ 5.67 (s, 1H, C**H**_2_=C); 5.33 (s, 1H, C**H**_2_=C), 3.78 (m, 4H, −C**H**_2_–COOH), 3.78 (m, 1H, −C**H**–COOH), 3.34 (t, 2H, NH–C**H**_2_–CH_2_), 1.94 (s, 3H, C**H**_3_–C), 1.5 (m, 6H, C–C**H**_2_–C) ppm. ^13^C NMR (D_2_O): δ 172.50 (COOH, NTA carboxylates), 170.28 (C=O, amide carbonyl), 139.27 (Cq, vinyl carbon of MA), 120.68 (CH_2_=C, vinyl), 68.08 (−CH_2_–N–COOH, NTA α-carbon), 56.28 (−N–CH_2_–COOH, methylenes next to tertiary N), 38.99 (−CH_2_–NH–, linker), 28.04 and 23.53 (−CH_2_–CH_2_–, internal aliphatic methylenes), 17.70 (CH_3_–C= of methacrylamide).

### Membranised Complex Coacervate Formation

Q-Am-MA and CM-Am-MA were dissolved separately in 1× PBS. Coacervation was induced by adding Q-Am-MA (54 μL, 1 mg/mL) to CM-Am-MA (46 μL, 1 mg/mL) in molar charge ratios Q-Am-MA:CM-Am-MA 2:1 while shaking at 1400 rpm at 21 °C, in an Eppendorf ThermoMixer. After 1 min, LAP photoinitiator (20 μL 3 mg/mL solution in PBS) was added, and after 5 min, terpolymer (3 μL, 25 mg/mL in DMSO) was added and shaken for an additional 10 s. When using NTA-MA, 2 μL (15 mg/mL in 1X PBS) NTA-MA and 0.5 μL Ni_2_SO_4_ (0.3 M) were added after 1 min of mixing Q-Am-MA and CM-Am-MA during formulation. A stability study was conducted using increasing concentrations of LAP or NTA-MA, as determined by brightfield optical microscopy (Figure S12). Acryloxyethyl thiocarbamoyl rhodamine B was added (1 μL, 1 mg/mL in DMSO) before the terpolymer stabilization for 3DPR samples to show the 3D distribution of the network within the coacervates.

### 3DPR Printing

Cross-linking of assembled complex coacervate artificial cells was performed on bulk coacervate samples in Ibidi microscopy slides (100 μL volumes in 18-well slides), using 365 nm UV light (CoolLED pE-800 device, 50 mW/mm^2^), irradiated homogeneously from a 10 cm distance to a 25 mm × 35 mm illumination area, to create cross-linked coacervate droplets. Laser intensity was kept constant at 100%, and the effect of cross-linking time was investigated; a size distribution is shown in Figure S13.

When cross-linking artificial cells in a spatially defined 3DPR, the 405 nm UV laser of the Leica TCS SP8 CLSM was used (Diode laser, 50 mW/mm^2^). 3DPRs of interest were drawn using the Leica LASX software and irradiated at 100% laser intensity for varying times in the focal plane of the 63×/0.5 NA water objective.

### Uptake Assays

Cargo uptake in artificial cells was studied by adding fluorophores to the supernatant. Coacervate artificial cells were prepared as described above and bulk cross-linked for 1, 5, and 10 min, under a UV lamp at 100% intensity. Fluorescein-based fluorophores (carboxyfluorescein (CF), FITC-4 kDa dextran (FITC-Dex 4 kDa), FITC-40 kDa dextran (FITC-Dex 40 kDa), and FITC-150 kDa dextran (FITC-Dex 150 kDa)) were added, 100 μL of each sample was transferred to an 18-well glass bottom microscope slide, and imaged after a 5 min incubation period, equal for all samples. Internal coacervate fluorescence was quantified with ImageJ. Partitioning coefficients (*K*_p_) were calculated from the following equation (eq 1), where *I*_coacervate_ is the internal coacervate fluorescence, *I*_dilute phase_ is the external fluorescence signal, and *I*_background_ is the background signal of the buffer (PBS).^40^

1 $$K_{p}=\left(I_{coacervate}-I_{background}\right)/\left(I_{dilute\;phase}-I_{background}\right)$$

Localized protein uptake was also evaluated using confocal microscopy. Coacervates were formed, and a 3DPR was fabricated in a selected artificial cell with 405 nm laser irradiation. His_6_-tagged mTurquoise protein was added to the artificial cell supernatant, and uptake was evaluated using confocal microscopy.

In addition, sequential printing of 2 separate 3DPRs and subsequent cargo uptake behavior was studied. Coacervates were formed, and the first 3DPR was fabricated in multiple artificial cells with 405 nm laser irradiation. Upon Cy5-β-Gal-His_6_ addition (the first cargo), the sample was incubated in a dark, sealed environment overnight. The second 3DPR was fabricated in close proximity to the first 3DPR, but in a different shape. Following, His_6_-tagged mTurquoise (the second cargo) was added, and the samples were incubated in a dark, sealed environment overnight. The uptake behavior was measured both after the first cargo addition, and the second cargo addition.

### Statistical Analyses

For the uptake assays, normality of residuals was tested by the Shapiro–Wilk test. To assess the individual effects of molecular weight and cross-linking time on cargo uptake dynamics, Kruskal–Wallis tests were performed, followed by Dunn’s post hoc analysis for multiple comparisons. Finally, to evaluate the combined effects of molecular weight and cross-linking time, a two-way ANOVA test with the Geisser-Greenhouse correction was performed, as the data set exhibited minor violations of the sphericity assumptions. Tukey’s multiple comparisons test was applied for post hoc analysis of pairwise group differences. A threshold of *p* < 0.05 was considered statistically significant for all measurements. A total of 35 individual coacervate droplets were analyzed across 3 experiments conducted independently by using different samples.

For the sequential printing and cargo uptake experiments, the normality of residuals was tested by the Shapiro–Wilk test. To compare the cargo uptake efficiencies across printing steps, a repeated-measures two-way ANOVA test with the Geisser-Greenhouse correction was performed, as the data set exhibited minor violations of the sphericity assumptions. Tukey’s multiple comparisons test was applied for post hoc analysis of pairwise group differences. A threshold of *p* < 0.05 was considered statistically significant for all measurements. Fluorescence intensity measurements were recorded from two coacervates and two 3DPRs within each coacervate. Randomly selected five regions of interest (ROIs) point measurements were recorded per 3DPR.

Statistical analyses were performed using GraphPad Prism 10.6.1.

### Fluorescence Recovery after Photobleaching (FRAP)

Coacervate artificial cells were prepared as described above and loaded with 250 nM of fluorescein-based fluorophore (FITC-4 kDa dextran). 100 μL sample was transferred to an 18-well glass-bottom microscope slide (Ibidi), and different samples were batch irradiated for different times. FRAP experiments were performed using the FRAP interface available in Leica LASX software. An initial image was acquired to define the region of interest (ROI), 5–10 μm in diameter, within a coacervate. Subsequently, the ROI was bleached by 3 iterations of 488 nm, 100% laser power. The recovery was monitored with a 1 sec interval. The intensities of the bleached ROI, reference area, a nearby coacervate that was not bleached, and background were extracted from the images using FIJI (ImageJ). The data were normalized by subtracting the background intensity and then dividing by the intensity of the reference area. A first-order exponential equation was fitted using Origin 2020 (OriginLab), from which the immobile fraction and recovery half-life were calculated as reported (fittings shown in Figure S14).^41,42^ The immobile fraction (*IM*_f_) of the fluorophore was calculated using the following equation (eq 2), where *I*_plat._ is the fluorescence intensity at the recovery plateau, *I*_0_, is the bleached fluorescence intensity, and *I*_i_ is the initial fluorescence intensity.

2 $$IM_{f}=1-(\left(I_{plat.}-I_{0}\right)/\left(I_{i}-I_{0}\right)$$

The diffusion constants (*D*) were calculated from eq 3, where ω is the radius of the bleached spot, and τ_1/2_ is the fluorescence recovery half-time. τ_1/2_ was calculated from eq 4, where τ is the fluorescence recovery time constant.

3 $$D=(\omega^{2}{\left(0.225\right))/\tau }_{1/2}$$

4 $$\tau_{1/2}=\tau \ln\left(2\right)$$

### Compartmentalized Enzyme Assay

Localized enzyme activity was evaluated using confocal microscopy. Coacervates were formed as described above, and a 3DPR was fabricated in a selected artificial cell with 405 nm laser irradiation. The His_6_-tagged β-Gal enzyme was added to the artificial cell supernatant (5 μL, 1 mg/mL). Technical triplicates of enzyme-loaded coacervates were made as described above. Following this, 1 μL of a pro-fluorescent substrate, fluorescein Di-β-d-galactopyranoside (FDG) (final concentration 250 μM), was added. Product formation was monitored for over 30 min, and measurements were taken every 30 s, recording the fluorescein emission wavelength.

## Supplementary Material



## Notes

Bulk cross-linked coacervate stability over different irradiation times, cargo diffusivity over different irradiation times, two-way ANOVA analysis tables for the cross-linking time and molecular weights effects on cargo uptake behavior, 3D projections of the 3DPR, fluorescence intensity measurements of His_6_-mTurquoise uptake and enzymatic reaction on 3DPR, ^1^H NMR spectrum of Q- and CM-amylose modifications, ^1^H NMR spectrum of -MA modifications of Q- and CM-amyloses, ^13^C NMR spectrum of NTA-MA, reaction schematics for the synthesis protocols, and coacervate structural stability in the presence of different concentrations of photoinitiator, and NTA-MA (PDF)
