# Supplementary material for: Intracompartmental 3D Printing of Enzymatically Active Organelle Mimics
Source: ACS Nano. 2025 Nov 6;19(45):39327–36. doi: 10.1021/acsnano.5c14167 (PMC12632173; doi:10.1021/acsnano.5c14167)
Supplement: Supplementary file 1 [file nn5c14167_si_001.pdf]

Supporting Information:

Intracompartmental 3D Printing of Enzymatically Active Organelle Mimics

Yiğitcan Sümbelli[1], Anna C. Jäkel[3], Madelief A. M. Verwiel[1], Nadia A. Erkamp[1], Alexander F. Mason[4], Friedrich C. Simmel[3], Jan C.M. van Hest[1, 2,] *, Alexander B. Cook[2,] *

[1]Department of Biomedical Engineering, Institute for Complex Molecular Systems (ICMS), Eindhoven University of Technology, Eindhoven, 5600 MB, Netherlands

[2]Department of Chemical Engineering and Chemistry, Institute for Complex Molecular Systems (ICMS), Eindhoven University of Technology, Eindhoven, 5600 MB, Netherlands

[3]Physics of Synthetic Biological Systems, Department of Bioscience, School of Natural Sciences, Technical University of Munich, Garching, 85748, Germany

[4]School of Science, Molecular Horizons, University of Wollongong, Wollongong, NSW 2522, Australia

* Corresponding authors: j.c.m.v.hest@tue.nl and a.b.cook@tue.nl

Contents

Figure S1. Brightfield optical microscopy images of coacervate samples, as an assessment of impact of crosslinking and UV light on artificial cells. 4

Figure S2. Quantification of droplet size through microscopy image analysis.................................. 4

Figure S3. CLSM micrographs showing the necessity of terpolymer stabilization despite the microgel formation due to bulk irradiation. 4

Figure S4. Fluorescence recovery after photobleaching (FRAP) experiments, showing the effect of crosslinking degree on FITC-Dex (4 kDa) diffusion through the crowded droplets. 5

Figure S5. Fluorescence recovery after photobleaching (FRAP) to determine FITCDex (4kDa) diffusion, with fitting model equation and obtained parameter values, of the methacrylated coacervate system studied in this article, with differing crosslinking degrees. 5

Figure S6. Diffusion constant calculations for FITC-Dex (4 kDa) cargo dynamicity within bulk crosslinked coacervates. Samples were irradiated for different times, separately............................ 6

Figure S7. CLSM micrographs of multiple 3DPR printing within a population of photopolymerizable coacervates. 7

Figure S8. 3D projections of a printed pattern within a photopolymerizable coacervate. 7

Figure S9. Localization of His_6-tagged fluorescent proteins in the organelle region after a 4 h incubation period.. 7

Figure S10. Fluorescence intensity quantification shows that the reaction rate is significantly higher in NTA-containing 3DPRs than in regions without NTA 8

Figure S11. ^1H nuclear magnetic resonance spectrum of quaternised amine functional amylose polymer, Q-Am, in deuterated water. 8

Figure S12. ^1H nuclear magnetic resonance spectrum of carboxymethyl functional amylose polymer, CM-Am, in deuterated water.. 9

Figure S13. Reaction scheme for the synthesis of quaternised amine functional amylose polymer, Q-Am, and subsequent modification to give Q-Am-MA... 9

Figure S14. Reaction scheme for the synthesis of carboxymethyl functional amylose polymer, CM-Am, and subsequent modification to give CM-Am-MA. 9

Figure S15. ^1H nuclear magnetic resonance spectrum of quaternised amine functional amylose polymer, after methacrylation, Q-Am-MA, in deuterated water. 10

Figure S16. ^1H nuclear magnetic resonance spectrum of carboxymethyl functional amylose polymer, after methacrylation, CM-Am-MA, in deuterated water. 10

Figure S17. Reaction scheme for the synthesis of monomer NTA methacrylamide, NTA-MA, 2,2'-((1-carboxy-5-methacrylamidopentyl)azanediyl)diacetic acid. 10

Figure S18. ^1H nuclear magnetic resonance spectrum of synthesised monomer NTA methacrylamide, NTA-MA, 2,2'-((1-carboxy-5-methacrylamidopentyl)azanediyl)diacetic acid in deuterated water... 11

Figure S19. ^{13}C nuclear magnetic resonance spectrum of synthesised monomer NTA methacrylamide, NTA-MA, 2,2'-((1-carboxy-5-methacrylamidopentyl)azanediyl)diacetic acid in deuterated water... 11

Figure S20. Brightfield optical microscopy images to assess coacervate stability in increasing concentrations of charged small molecules 12

Table S1. Two-way ANOVA analysis table for the relationship between the effects of crosslinking time and molecular weight on the partitioning of cargo molecules. 6

Table S2. Two-way ANOVA analysis table for the source of variation between the effects of crosslinking time and molecular weight on the partitioning of cargo molecules 6

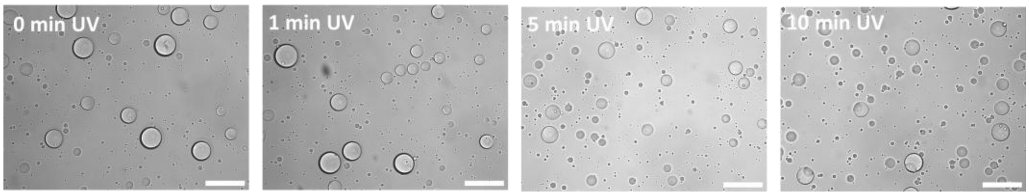

Figure S1. *Brightfield optical microscopy images of coacervate samples, as an assessment of impact of crosslinking and UV light on artificial cells. Membranised coacervate artificial cells formed and crosslinked in bulk following the procedure described in the materials and methods section. Images same dimensions, scale bars 100 µm.*

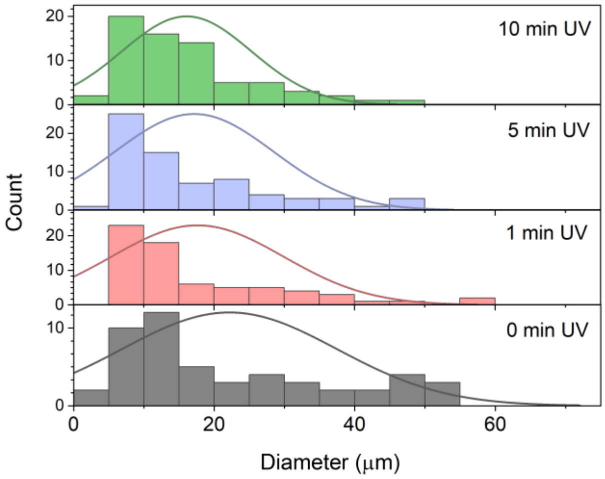

Figure S2. *Quantification of droplet size (diameter) through microscopy image analysis, n >60 droplets per sample. Membranised coacervate artificial cells formed and crosslinked in bulk following the procedure described in the materials and methods section.*

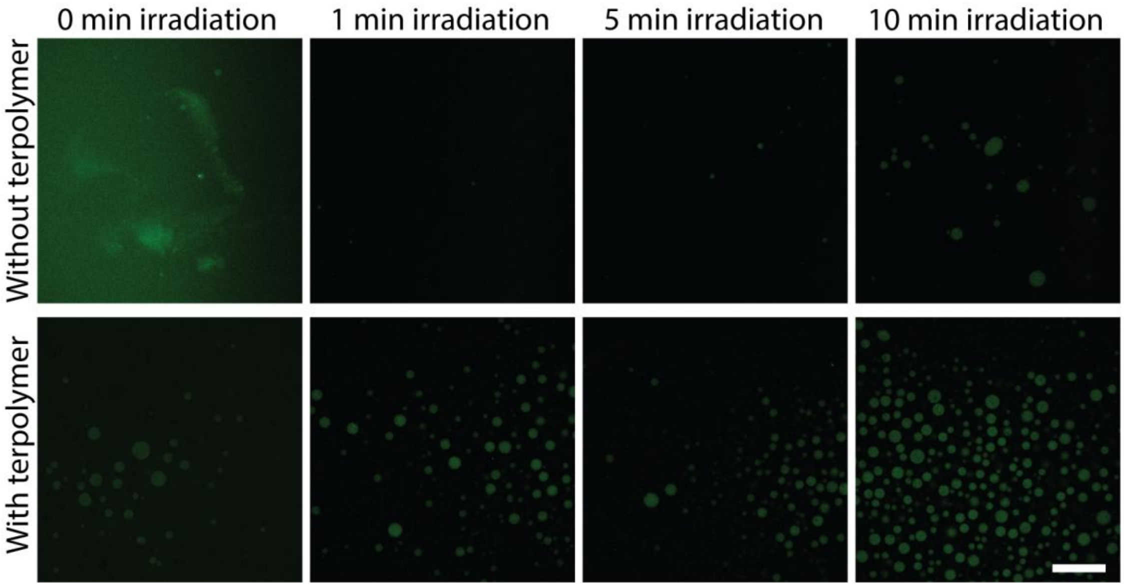

Figure S3. *CLSM micrographs showing the necessity of terpolymer stabilization despite the microgel formation due to bulk irradiation. (Amylose component is labeled with Cyanine 3) (Scale bar: 100 µm).*

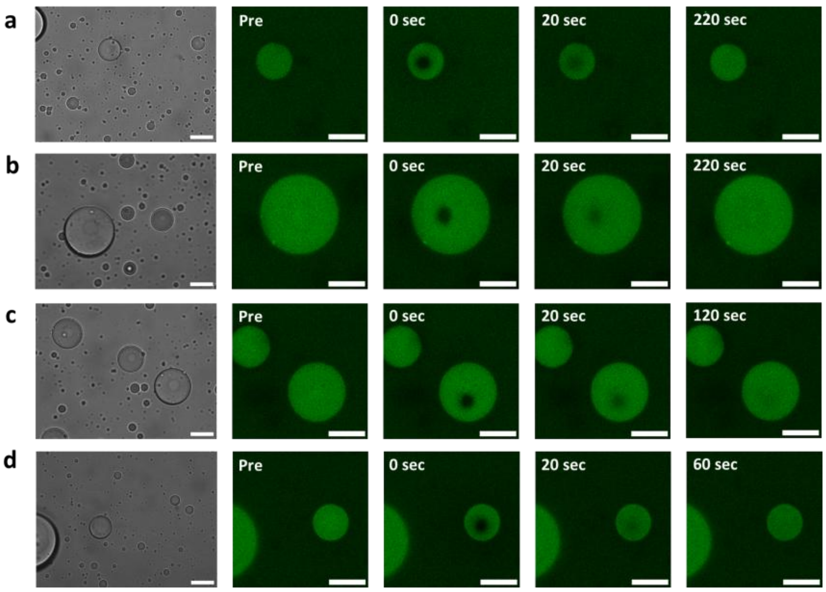

Figure S4. *Fluorescence recovery after photobleaching (FRAP) experiments, showing the effect of crosslinking degree on FITC-Dex (4 kDa) diffusion through the crowded droplets. Membranised coacervate artificial cells formed and crosslinked in bulk following the procedure described in the materials and methods section, a) 10 min UV light. b) 5 min UV light, c) 1 min UV light, d) 0 min UV light. (Scale bars: 20 µm)*

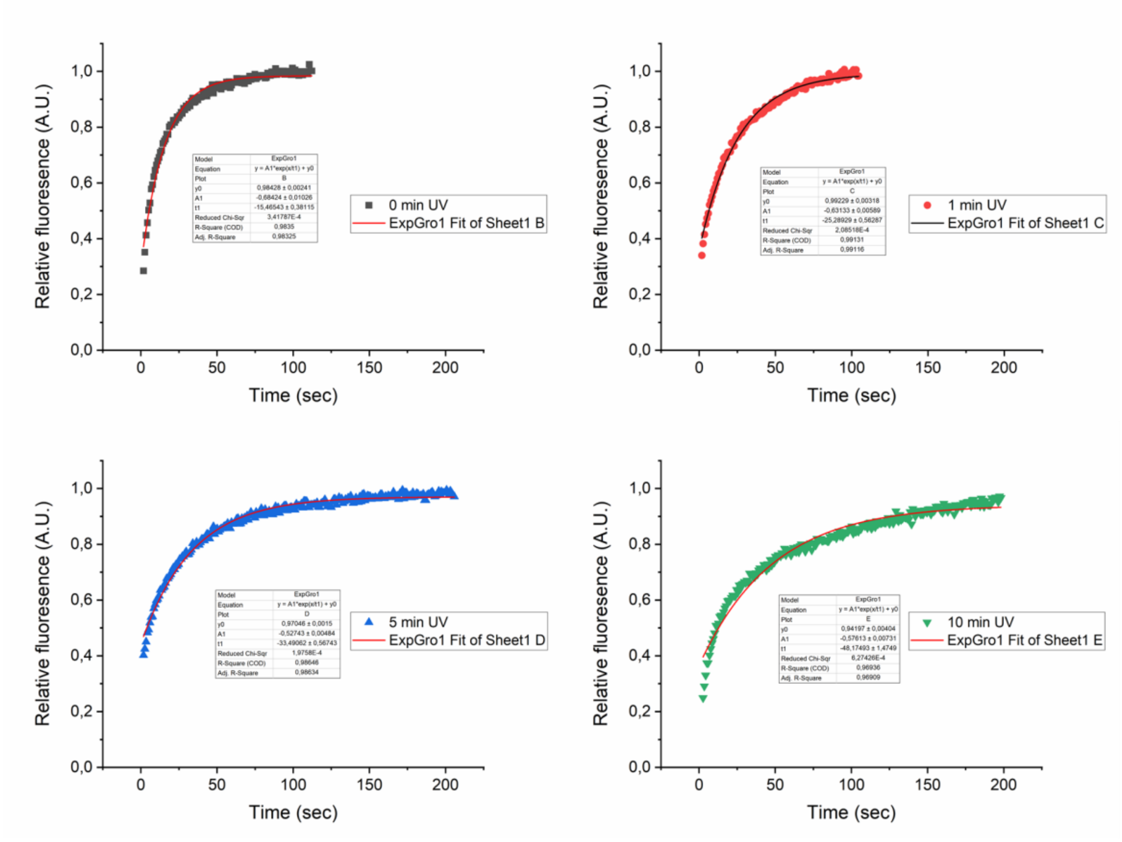

Figure S5. *Fluorescence recovery after photobleaching (FRAP) to determine FITCDex (4kDa) diffusion, with fitting model equation and obtained parameter values, of the methacrylated coacervate system studied in this article, with differing crosslinking degrees.*

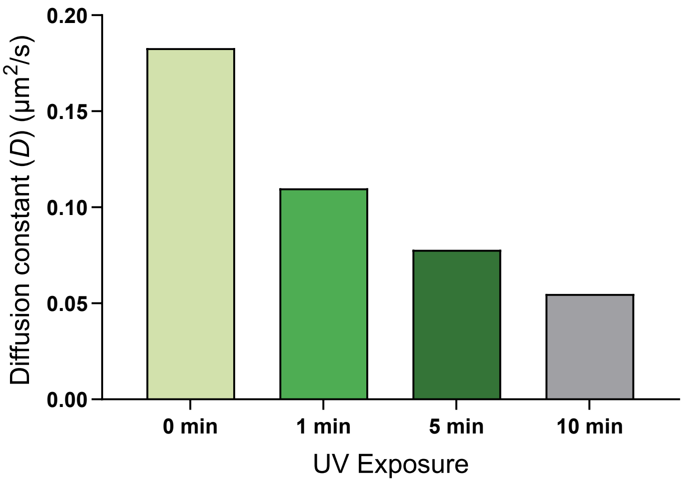

Figure S6. Diffusion constant calculations for FITC-Dex (4 kDa) cargo dynamicity within bulk crosslinked coacervates. Samples were irradiated for different times, separately.

Table S1. *Two-way ANOVA analysis table for the relationship between the effects of crosslinking time and molecular weight on the partitioning of cargo molecules. A higher F-value for molecular weight indicates the dominant influence on partitioning. High SS and F-values also show the significance of the combined effect of molecular weight and crosslinking time. (SS: sum of squares, DF: degree of freedom, MS: mean squares, DFn: degree of freedom of numerator, DFd: degree of freedom of denominator)*

F-statistics Table	*SS*	*DF*	*MS*	*F(DFn, DFd)*	*P value*
Crosslinking time x Molecular weight	50.04	9	5.560	F (6.664, 293.2) = 17.86	P<0.0001
Crosslinking time	7.810	3	2.603	F (2.221, 293.2) = 8.361	P=0.0002
Molecular weight	2477	3	825.7	F (3, 132) = 484.4	P<0.0001
Subject	225.0	132	1.705	F (132, 396) = 5.475	P<0.0001
Residual	123.3	396	0.3114		

Table S2. *Two-way ANOVA analysis table for the source of variation between the effects of crosslinking time and molecular weight on the partitioning of cargo molecules*

Source of Variation	*% of total variation*	*P value*	*P-value summary*	*Significant?*	*$\varepsilon_{Geisser\text{-}Greenhouse}$*
Crosslinking time x Molecular weight	1.736	<0.0001	****	Yes	0.7405
Crosslinking time	0.2709	0.0002	***	Yes	0.7405
Molecular weight	85.91	<0.0001	****	Yes	
Subject	7.804	<0.0001	****	Yes	

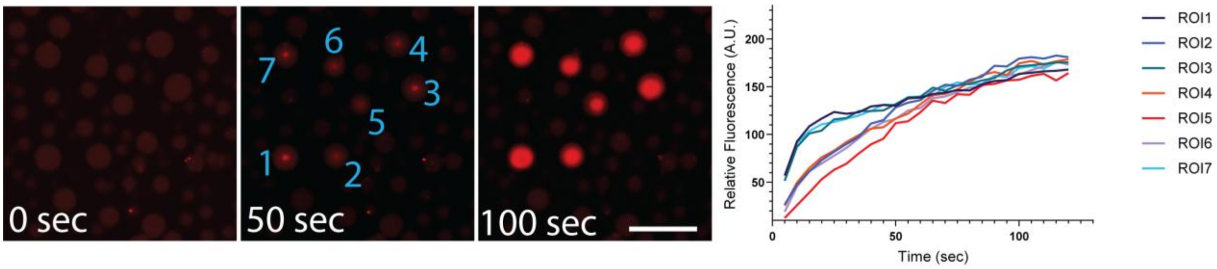

Figure S7. CLSM micrographs of multiple 3DPR printing within a population of photopolymerizable coacervates. The 3DPR emits fluorescent signal due to the polymerization of photopolymerizable dye, acryloxyethyl thiocarbamoyl rhodamine B. The signal increase can be observed throughout the printing process (Scale bar: 50 µm).

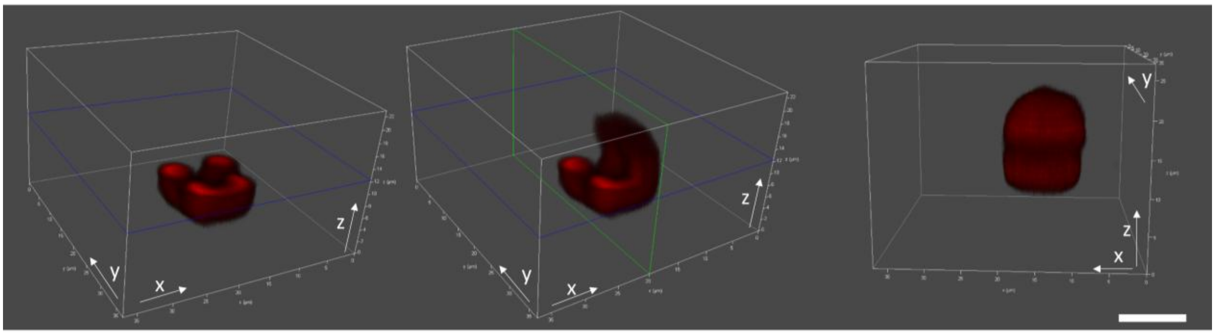

Figure S8. 3D projections of a printed pattern within a photopolymerizable coacervate. The 3DPR emits fluorescent signal due to the polymerization of photopolymerizable dye, acryloxyethyl thiocarbamoyl rhodamine B. The projections were digitally created by using z-stacks of the sample with CLSM. (Scale bar: 10 µm, slice thickness of the z-stacks: 0.5 µm)

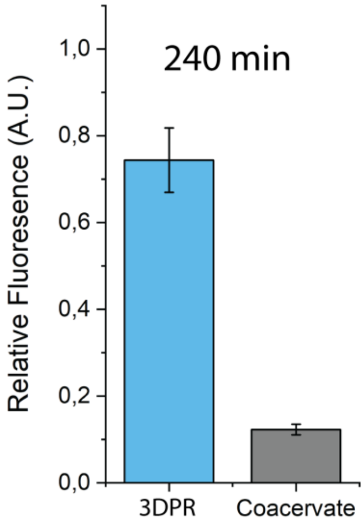

Figure S9. Localization of His_6-tagged fluorescent proteins in the organelle region after a 4 h incubation period

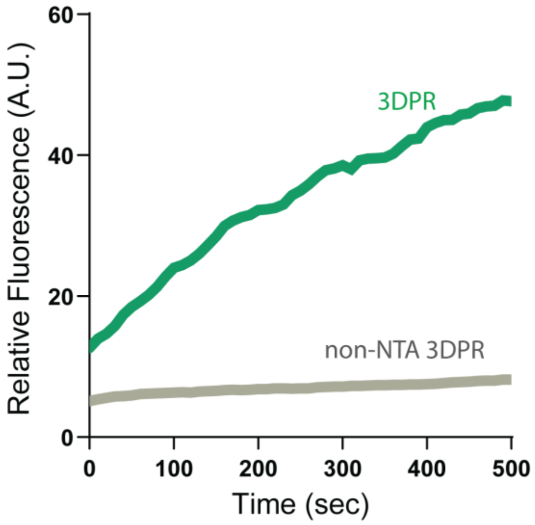

Figure S10. Fluorescence intensity quantification shows that the reaction rate is significantly higher in NTA-containing 3DPRs than in regions without NTA

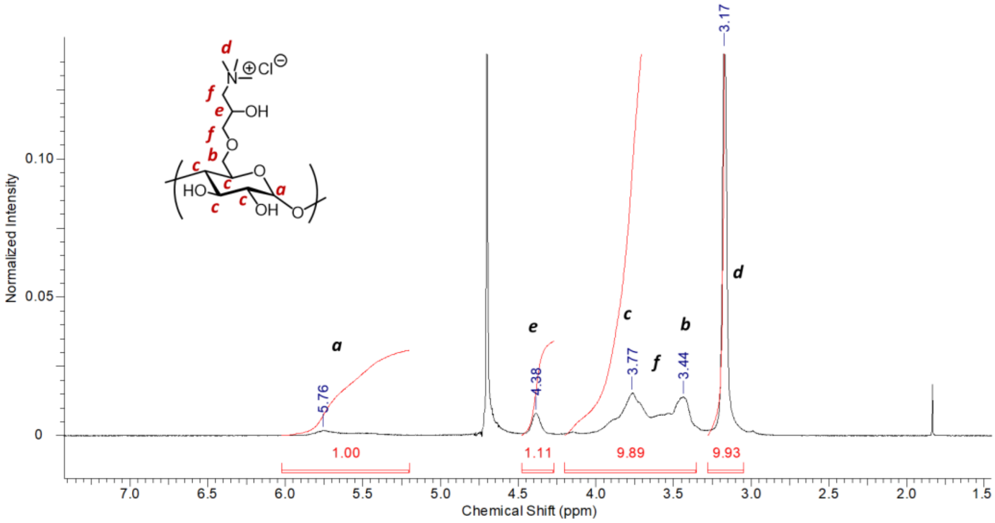

Figure S11. *^{1}H nuclear magnetic resonance spectrum of quaternised amine functional amylose polymer, Q-Am, in deuterated water.*

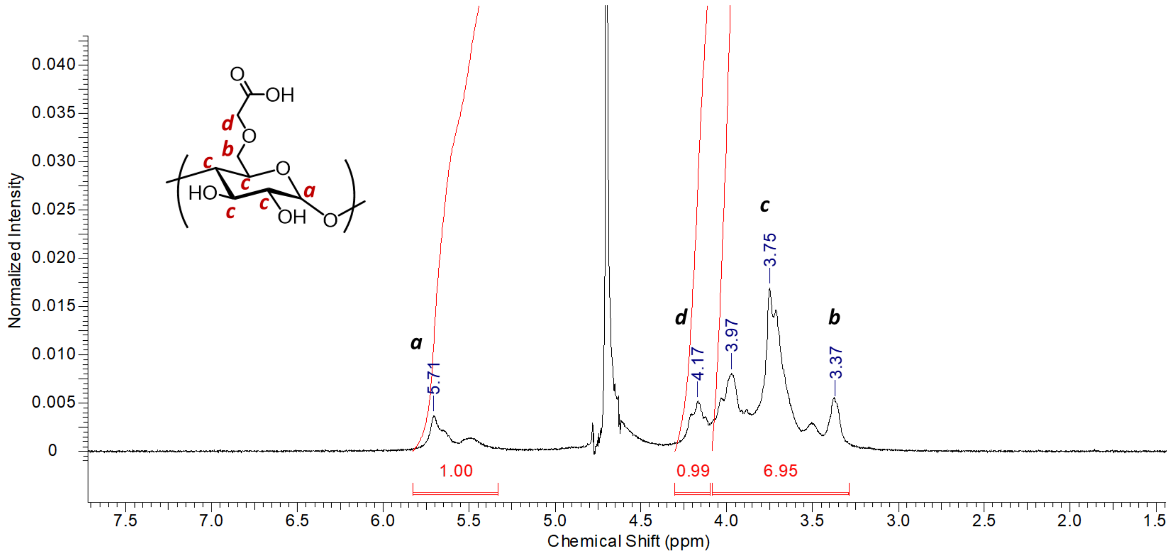

Figure S12. *^{1}H nuclear magnetic resonance spectrum of carboxymethyl functional amylose polymer, CM-Am, in deuterated water.*

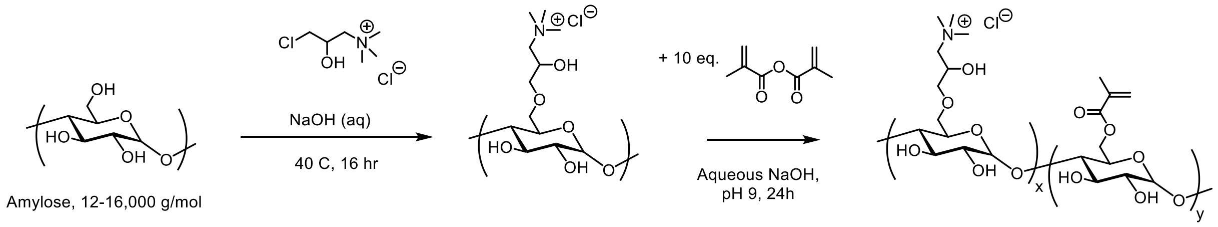

Figure S13. *Reaction scheme for the synthesis of quaternised amine functional amylose polymer, Q-Am, and subsequent modification to give Q-Am-MA.*

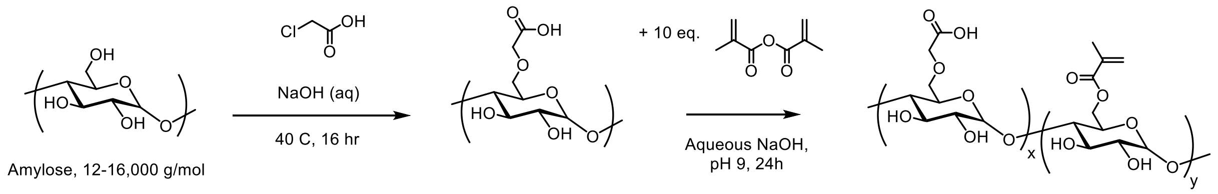

Figure S14. *Reaction scheme for the synthesis of carboxymethyl functional amylose polymer, CM-Am, and subsequent modification to give CM-Am-MA.*

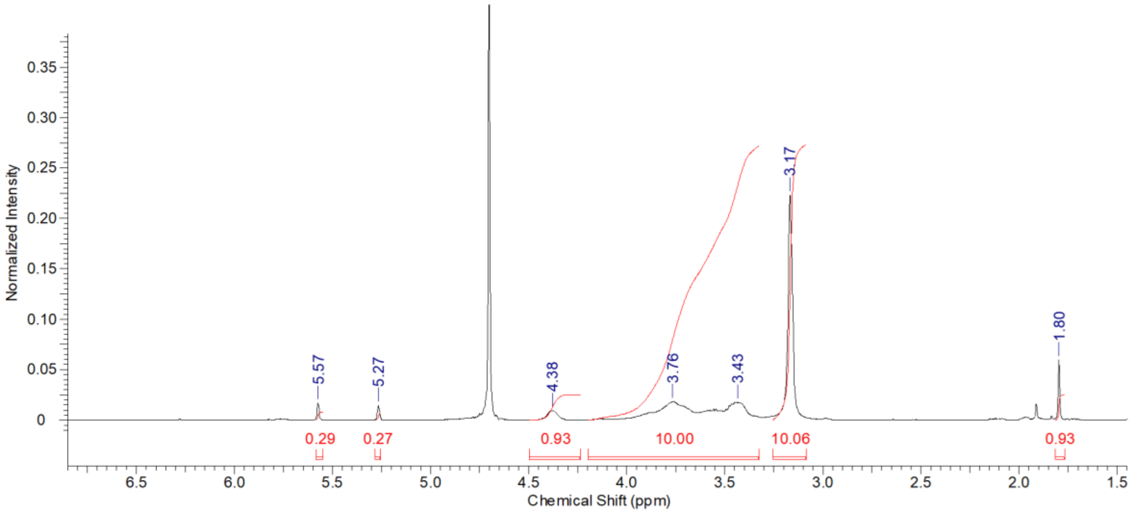

Figure S15. *^{1}H nuclear magnetic resonance spectrum of quaternised amine functional amylose polymer, after methacrylation, Q-Am-MA, in deuterated water.*

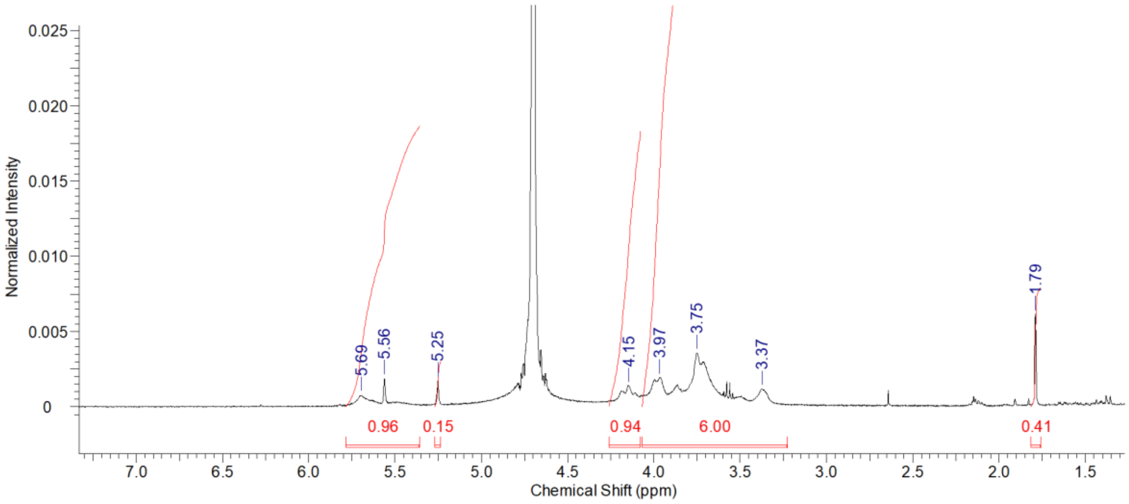

Figure S16. *^{1}H nuclear magnetic resonance spectrum of carboxymethyl functional amylose polymer, after methacrylation, CM-Am-MA, in deuterated water.*

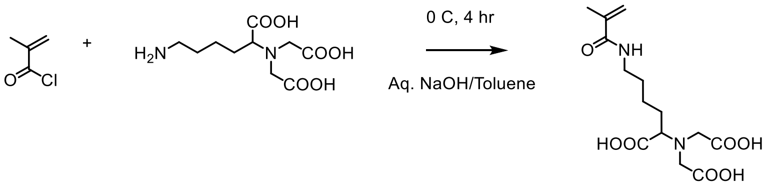

Figure S17. *Reaction scheme for the synthesis of monomer NTA methacrylamide, NTA-MA, 2,2'-((1-carboxy-5-methacrylamidopentyl)azanediyl)diacetic acid.*

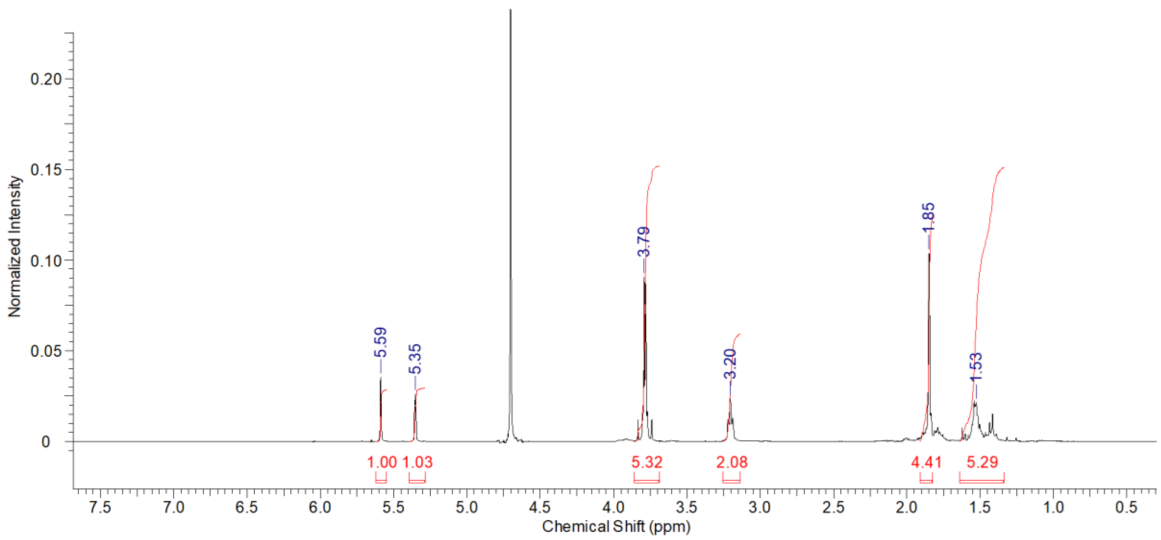

Figure S18. *^{1}H nuclear magnetic resonance spectrum of synthesised monomer NTA methacrylamide, NTA-MA, 2,2'-((1-carboxy-5-methacrylamidopentyl)azanediyl)diacetic acid in deuterated water.*

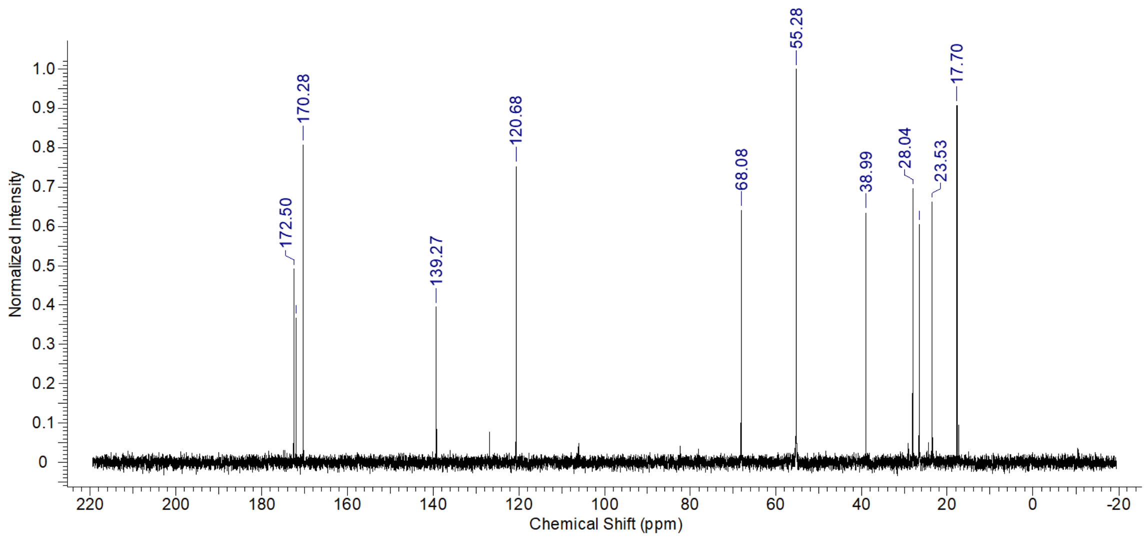

Figure S19. *^{13}C nuclear magnetic resonance spectrum of synthesised monomer NTA methacrylamide, NTA-MA, 2,2'-((1-carboxy-5-methacrylamidopentyl)azanediyl)diacetic acid in deuterated water.*

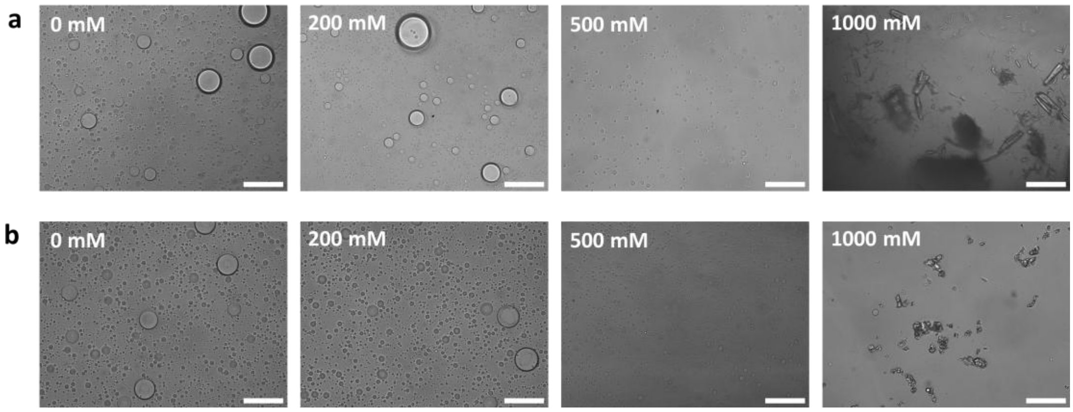

Figure S20. *Brightfield optical microscopy images to assess coacervate stability in presence of increasing concentrations of charged small molecules a) LAP photoinitiator and b) NTA-MA. Membranised coacervate artificial cells formed without UV irradiation, and with different NTA-MA and LAP concentrations. Images same dimensions, (Scale bars: 100 µm)*
